# PLA/PMMA Reactive Blending in the Presence of MgO as an Exchange Reaction Catalyst

**DOI:** 10.3390/polym17070845

**Published:** 2025-03-21

**Authors:** Masoud Komeijani, Naeimeh Bahri-Laleh, Zohreh Mirjafary, Massimo Christian D’Alterio, Morteza Rouhani, Hossein Sakhaeinia, Amin Hedayati Moghaddam, Seyed Amin Mirmohammadi, Albert Poater

**Affiliations:** 1 Department of Chemistry, Science and Research Branch, Islamic Azad University, Tehran 1477893855, Iran; mskomman@gmail.com (M.K.); zmirjafary@srbiau.ac.ir (Z.M.); morteza.rouhani@alumni.znu.ac.ir (M.R.); 2 International Institute for Sustainability with Knotted Chiral Meta Matter (WPI-SKCM2), Hiroshima University, 1-3-1 Kagamiyama, Higashi-Hiroshima, Hiroshima 739-8526, Japan; nbahri@hiroshima-u.ac.jp; 3 Dipartimento di Scienze Chimiche, Università di Napoli Federico II, Complesso Monte S. Angelo, Via Cintia, I-80126 Napoli, Italy; massimochristian.dalterio@unina.it; 4 Department of Chemical Engineering, Central Tehran Branch, Islamic Azad University, Tehran 1496969191, Iran; h.sakhaeinia@iauctb.ac.ir (H.S.); ami.hedayati_moghaddam@iauctb.ac.ir (A.H.M.); 5 Institut de Química Computacional i Catàlisi and Departament de Química, Universitat de Girona, c/Maria Aurèlia Capmany 69, 17003 Girona, Spain

**Keywords:** poly(lactic acid), poly(methyl methacrylate), reactive blending, transesterification reaction, MgO nanoparticle, cell culture

## Abstract

To address the limitations of poly (lactic acid) (PLA), it was blended with poly (methyl methacrylate) (PMMA) as a toughening component, using MgO nanoparticles (NPs, 0.075–0.15 wt%) as a catalyst. SEM pictures confirmed the good miscibility of the blends. Mechanical tests showed a slight decrease in elastic modulus and tensile strength for the PLA/PMMA125 sample containing 0.125% MgO. Yet, elongation at break rose by over 60% and impact strength increased by over 400% compared to pure PLA. Also, MgO facilitated the shifting of the glass transition temperature (T_g_) of both polymers in DSC curves. Additionally, the absence of cold crystallization in PLA, coupled with reductions in its melting temperature (T_m_) and crystallinity, were identified as critical factors contributing to improved miscibility within the reactive blend. Melt flow index (MFI) evaluation indicated a decrease in viscosity, while water contact angle measurements revealed an increase in polar groups on the surfaces of the MgO-containing samples. X-ray diffraction (XRD) and transmission electron microscopy (TEM) analyses confirmed the effective distribution and dispersion of NPs throughout the blend, along with a significant decrease in crystallinity. Moreover, DFT calculations were performed to better understand the role of MgO in the reaction. The findings offered key insights into the reaction mechanism, confirming that MgO plays a crucial role in facilitating the transesterification between PLA and PMMA. These findings underscore the enhanced performance of exchange reactions between the active groups of both polymers in the presence of MgO, leading to the formation of PLA-PMMA copolymers with superior miscibility and mechanical properties. Finally, a cell culture assay confirmed the blend’s non-toxicity, showing its versatile potential.

## 1. Introduction

In recent years, both academia and industry have emphasized the development of renewable energy sources [1,2,3], particularly biodegradable polymers [4,5,6], towards sustainable [7,8], actually compostable [9,10], and specifically lead polymers for green solutions [11]. Among these polymers, poly (lactic acid) (PLA) and its copolymers have emerged as promising candidates due to their cost-effective production from renewable resources [12,13,14]. Currently, PLA ranks as the second most widely used compostable plastic globally [5,15,16,17]. In detail, PLA is a biodegradable and compostable polymer derived from renewable resources such as corn starch or sugarcane [18,19,20]. It is widely used in packaging, disposable cutlery, and biomedical applications due to its biocompatibility and eco-friendly profile. However, the terms biodegradable and compostable are often used interchangeably [21], despite having distinct scientific meanings. Biodegradability refers to a material’s ability to break down into natural elements (such as water, carbon dioxide, and biomass) through microbial activity over time. PLA is biodegradable under specific conditions, such as high temperatures and microbial presence, but its degradation in natural environments (e.g., soil or marine conditions) is significantly slower than that of other biopolymers. On the other hand, compostability, a subset of biodegradability, requires a material to break down within a defined timeframe under industrial or home composting conditions, leaving no toxic residue. PLA is industrially compostable, meaning it degrades efficiently at temperatures above 55 °C with sufficient humidity and microbial activity. However, in home composting or natural environments, PLA degradation occurs much more slowly due to lower temperatures. Scientifically, PLA degradation involves hydrolysis, followed by microbial digestion of lactic acid monomers. Its behavior differs from that of traditional petroleum-based plastics, making it a promising yet condition-dependent sustainable alternative.

Compared to traditional petroleum-based plastics, PLA exhibits several limitations [22], including poor toughness, low elongation at break, cold crystallization behavior [23], low heat resistance [24], brittleness, and reduced melt viscosity. These drawbacks necessitate modifications [25], particularly to enhance its mechanical properties [26]. One effective strategy involves blending PLA with high-strength, tough polymers such as polyamides, polyesters [27], polycarbonate bisphenol A, polyethylene glycol, poly (methyl methacrylate) (PMMA) [28], polystyrene, ethylene vinyl acetate copolymer, polypropylene [29], poly(ethylene-co-glycidyl methacrylate) [30], and poly(vinyl acetate) [31,32,33]. Polymer blending combines two or more polymers to enhance physical, mechanical (mainly toughness), and other desired properties [34,35]. However, achieving a uniform and homogeneous matrix often poses challenges due to poor miscibility between polymer phases, arising from high interfacial tension and insufficient chain entanglements during blending [36,37].

To address this, a compatibilizer or catalyst is required to promote miscibility and interactions between phases. Reactive compatibilizers, such as zinc oxide, tin (II) ethylhexanoate, cobalt (II) acetylacetonate, aluminum chloride, and magnesium oxide (MgO), facilitate exchange reactions (e.g., transesterification) between polymer phases during reactive melt blending [36,38,39]. In a reactive blending process, exchange reactions can occur between polymers containing ester, amide, carbonate, and other groups, resulting in the formation of block copolymers [40] that act as compatibilizers for immiscible polymer phases [41]. When an appropriate catalyst is added, additional copolymers can be formed, further enhancing compatibility [42,43]. It has been reported that the presence of such catalysts can lead to the cleavage of polyesters, polycarbonates, polyamides, and other polymer chains, which then link with available active groups such as ester, amide, carbonate, carboxyl, hydroxyl, and anhydride groups [26,36,37,39].

Furthermore, it has been reported that the methyl ester group of PMMA, in the presence of an appropriate catalyst, can react with active groups in polyesters or polycarbonates, forming copolymer chains containing PMMA [38,44]. Here, this characteristic of PMMA is utilized to enhance the toughness of PLA. The selection of an exchange reaction catalyst is a critical consideration, as it influences the scission mechanisms and, consequently, the mechanical properties. Here, this characteristic of PMMA is utilized to improve the toughness of PLA. The selection of an exchange reaction catalyst is a critical consideration, as it affects the scission mechanisms and, consequently, the mechanical properties [45]. In this study, we introduce MgO as a novel catalyst for use in the melt blending of PLA and PMMA.

While reactive melt blending results in some exchange reactions between these two polymers, leading to limited miscibility, the presence of MgO nanoparticles (NPs) significantly enhances miscibility. In addition to their catalytic properties, MgO NPs improve the overall properties of the resulting alloy. The analyses performed indicate that, compared to neat PLA, the fabricated blend exhibits distinct properties in the presence of the catalyst. Moreover, a biocompatibility (cell culture) test confirmed that the components of this alloy are not toxic.

## 2. Experimental Materials

### 2.1. Materials and Sample Preparation

PLA granules (Mw~200 kDa, PDI~1.8, and density 1.24 g/mL) were supplied by NatureWorks Co., Plymouth, MN, USA. PMMA pellets, grade V 825T, with a melt flow index (MFI) of 1.8 g/10 min (230 °C, 2.16 kg), were obtained from ALTUGLAS Co., Bristol, PA, USA. MgO nanoparticles (purity ≥ 99%) with a size range of 10–30 nm were provided by Nanosadra Co., Tehran, Iran. PLA and PMMA (70/30 wt%) were physically mixed and conditioned at 40 °C in a vacuum oven for 24 h. The mixture was then melt-blended with and without varying amounts of MgO nanoparticles using a twin-screw internal mixer (Brabender GmbH & Co., Duisburg, Germany). The mixer parameters were set to a capacity of 60 mL, screw speed of 80 rpm, temperature of 195 °C, and residence time of 7 min. The resulting compounds were thermoformed into dumbbell-shaped and notched rod specimens, each with a thickness of 3 mm, for tensile and Izod impact testing, respectively. This was achieved using a Mini Test Press No. 519 (Toyoseiki Co., Tokyo, Japan). The compositions of the prepared samples are summarized in Table 1.

### 2.2. Characterization

Tensile and Izod tests were conducted to assess the mechanical properties of the samples. Tensile properties were evaluated using a universal tensile machine (SMT-150, Santam Co., Tehran, Iran) at a crosshead speed of 50 mm/min. The sample dimensions were 40 × 10 × 1 mm³. The tensile tests were conducted in accordance with the ASTM D638 standard [46]. Notched samples were subjected to Izod impact testing using an Izod analyzer (SIT-20E, Santam Co., Tehran, Iran), following ASTM D256 standards [47].

Thermal properties were analyzed using a PL-800 differential scanning calorimeter (DSC) (Polymer Laboratories Co., Shropshire, UK). Tests were conducted over a temperature range of 0–200 °C at a heating rate of 10 °C/min under a nitrogen atmosphere. To eliminate thermal history, each sample was held at 200 °C for 4 min at the end of the first heating cycle, and the results from the second heating cycle were reported.

The degree of crystallinity of the PLA in the blends was determined via the following equation:

X_c_ = [(∆H_m_ − ∆H_cc_)/Δ*H*^0^_m_] × 100 (1)

where ∆H_m_, ∆H_cc_, and Δ*H*^0^_m_ are the enthalpy of melting of the sample, the enthalpy of cold crystallization of the sample, and the enthalpy of melting of a perfect PLA crystal (93.6  J/g) [48].

Scanning electron microscopy (SEM) was employed to analyze the fracture surfaces. These surfaces were coated with gold in a low-pressure environment before being examined with a VEGA-TESCAN device, TESCAN Co., Brno, Czech Republic.

The melt flow index (MFI) of the prepared specimens was determined using a Zwick MFI analyzer (ZwickRoell Co., Ulm, Germany) following ASTM D1238 standards [49], at a temperature of 190 °C and loads of 2.16 and 5 kg. Surface hydrophilicity was evaluated using a G10 water contact angle (WCA) analyzer (Krüss Co., Hamburg, Germany). A microsyringe was used to deposit a 12 µL drop of distilled water onto the surface of each sample.

For all analyses, including tensile, Izod, MFI, and WCA, five replicates were tested, and the mean values were reported. X-ray diffraction (XRD) analysis was performed using a D5000 diffractometer (Siemens Co., Munich, Germany) within a 2θ range of 5–40° at a scan speed of 1.2°/min to investigate the crystalline structure of the polymer matrix and the dispersion of MgO nanoparticles (NPs) within the polymer phase. The dispersion of MgO NPs in the organic phase was further examined using an EM2085 transmission electron microscope (TEM, Philips Co., Amsterdam, The Netherlands) operating at 100 kV.

Statistical analysis was performed to validate the results. Multiple specimens (n > 3) were tested for each parameter, and results were reported as mean ± standard deviation. Statistical evaluations were conducted using MiniTab software (version 15), employing one-way analysis of variance (ANOVA). Results were considered statistically significant when the calculated *p*-value was less than 0.05.

### 2.3. Cell Culture Assay

To evaluate the in vitro biological activity and cytotoxicity of the prepared compounds, L929 mouse fibroblast cells (provided by the National Cell Bank of Iran, Pasteur Institute) were cultured on the specimens. The procedure was conducted following previously reported methods [33,50]. First, three pieces of each compound were sterilized with 70% (*v*/*v*) ethyl alcohol, exposed to UV irradiation, and washed in a culture medium. Next, a cell suspension with a concentration of 400,000 cells/mL was prepared using Roswell Park Memorial Institute (RPMI) 1640 medium. To enhance the medium, 10% (*v*/*v*) fetal bovine serum (FBS) and 100 mg/mL gentamycin were added as modifiers. The sterilized samples were then placed in a multi-well culture plate containing 5 mL of the cell suspension. For comparison, one well without a sample served as a negative control. The plate was incubated at 37 °C in a CO_2_ atmosphere for 72 h. After incubation, the samples were removed, washed with phosphate-buffered saline (PBS), and fixed for 24 h in a solution of 2.5% (*w*/*v*) glutaraldehyde (GA) in saline at 20 °C. Subsequently, the samples were dehydrated in a graded series of ethyl alcohol solutions (60%, 70%, 80%, and 95% *v*/*v*). The morphology of the adhered, stained cells was examined using a TE-2000-U Eclipse light microscope (Nikon Co., Tokyo, Japan). Additionally, the cytotoxicity of the compounds, or cell viability, was assessed using an MTT assay. For this, 100 µL of MTT solution (0.5 mg/mL) was added to each well, replacing 20% of the cell culture medium after 72 h of incubation. Following incubation, the medium was removed, and the resulting formazan crystals were dissolved in 150 µL of isopropyl alcohol for 20 min. The absorbance of each well was measured at 545 nm using a Stat Fax-2100 microplate reader (Awareness Co., Palm City, FL, USA), indicating the number of viable cells.

### 2.4. Quantum Mechanical Calculations

All Density Functional Theory (DFT) calculations were carried out using the Gaussian 16 software suite [51], with the B3LYP functional as formulated by Becke and Perdew [52,53,54]. The electronic structure was described using the SVP basis set, a standard split-valence basis set with a polarization function, developed by Ahlrichs and colleagues [55]. Stationary points were identified through vibrational analysis, which also provided zero-point energies and thermal corrections for enthalpy and entropy at 298.15 K and 1 bar. To improve electronic energy estimations, single-point energy calculations were performed with the 6–311G(d, p) basis set for all atoms [56], incorporating dispersion corrections, in detail, Grimme D3 correction with Becke–Johnson damping (EmpiricalDispersion = GD3BJ) [57] as implemented in Gaussian 16 and accounting for solvation effects via the PCM model with ethanol as the solvent [58]. Ethanol was selected to simulate the polar environment where polymeric chains are surrounded by -OH groups. The refined energies, along with the thermal corrections from the SVP level, are referred to as Gibbs energies in Δ*G*.

## 3. Results and Discussion

### 3.1. Mechanical Properties

**Figure 1:**
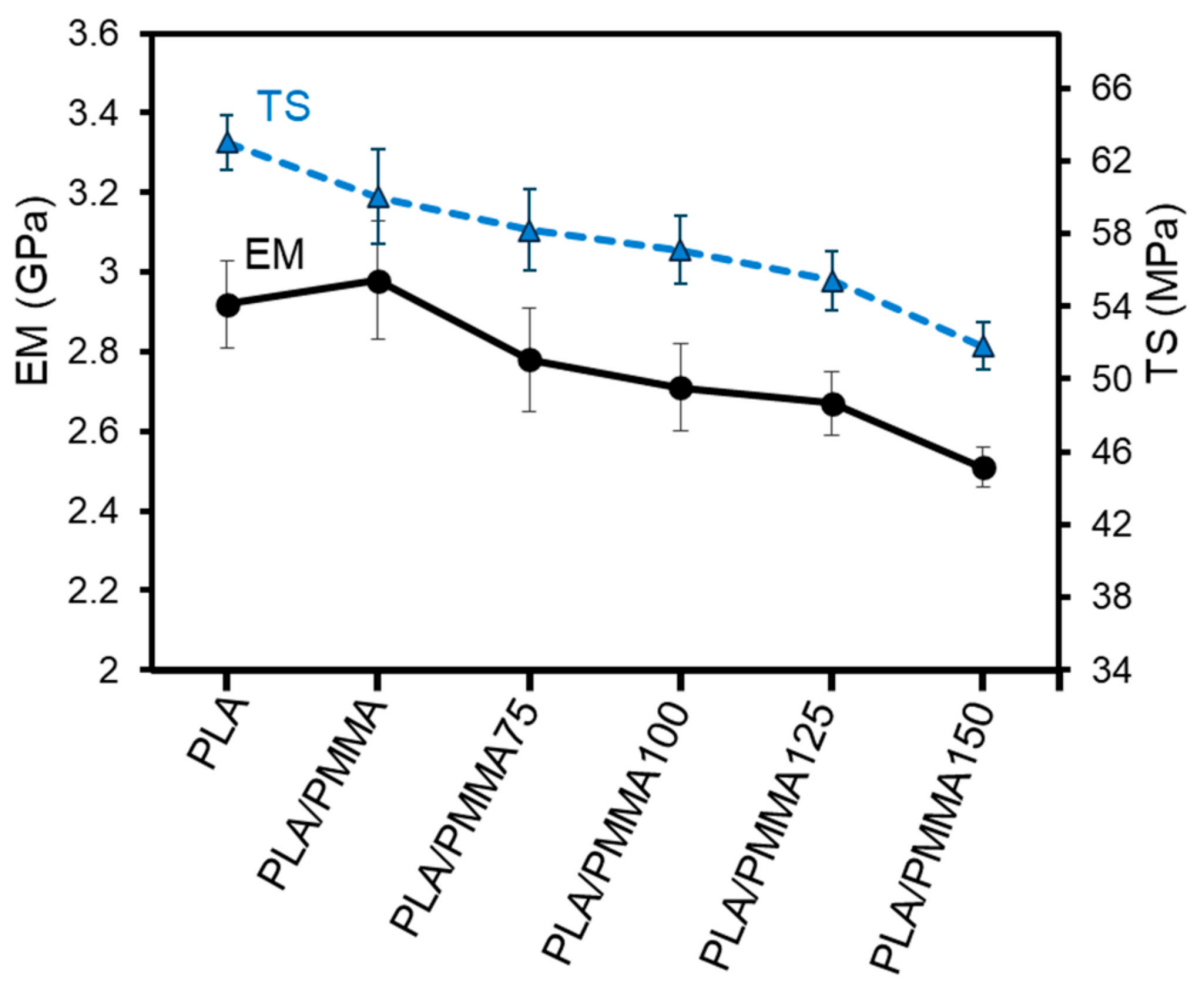
EM and TS trends for the fabricated samples.

Tensile testing is the most common destructive engineering test used to evaluate how a material withstands stress (tension) and to reveal its ductility, strength, and flexibility. This test provides key parameters such as Young’s (elastic) modulus (EM), ultimate tensile strength (TS), and elongation at break (EB). Figure 1 illustrates the trends for EM and TS values across the samples.

As shown in the EM curve, neat PLA has an EM of 2.92 GPa, which increases to 2.98 GPa when compounded with 30 wt% PMMA (sample PLA/PMMA). This increase is attributed to the higher modulus of PMMA, as no other factors appear to influence it. However, in the presence of 0.075 wt% MgO nanoparticles (NPs) in sample PLA/PMMA75, the EM value decreases to 2.78 GPa. This declining trend continues, with EM values of 2.71, 2.67, and 2.51 GPa observed for samples containing 0.1, 0.125, and 0.15 wt% MgO catalyst, respectively. This reduction in EM can be attributed to the catalytic role of MgO in facilitating exchange reactions between the reactive functional groups of PLA and PMMA. The PLA/PMMA blends typically undergo transesterification reactions, which will be discussed in detail in Section 3.6.

The statistical analysis of EM values shows that the differences between the samples are significant. Notably, the EM values for samples containing 0.125 wt% and 0.15 wt% MgO nanoparticles (NPs) differ significantly (*p* < 0.05). However, the differences between samples containing 0.075 and 0.1 wt% MgO NPs, as well as those containing 0.1 and 0.125 wt%, are not significant (*p* > 0.05). This indicates that the drop in EM values between PLA/PMMA125 and PLA/PMMA150 is pronounced, and in terms of modulus, the optimal MgO NP concentration is 0.125 wt%.

According to Figure 1, the TS of neat PLA decreases from 63 MPa to 60 MPa when blended with 30 wt% PMMA (sample PLA/PMMA). This reduction can be attributed to the lower TS of PMMA (58 MPa) and the poor miscibility between the two polymer phases. The downward trend continues more steeply with TS values of 58.2, 57.1, 55.4, and 51.8 MPa for samples containing 0.075, 0.1, 0.125, and 0.15 wt% MgO NPs, respectively.

Typically, changes in EM and TS parameters follow similar patterns for polymeric materials, as observed here. The decrease in TS values is likely due to the formation of PLA-PMMA copolymers, which reduce PLA crystallinity, vide infra. Statistical analysis confirms that the differences in TS values between all samples are significant. Furthermore, the difference between PLA/PMMA125 and PLA/PMMA150 samples is noteworthy (*p* < 0.05), whereas the difference between PLA/PMMA100 and PLA/PMMA125 samples is negligible (*p* > 0.05). Based on these results, the optimal MgO NP concentration for TS, like EM, is 0.125 wt%.

**Figure 2:**
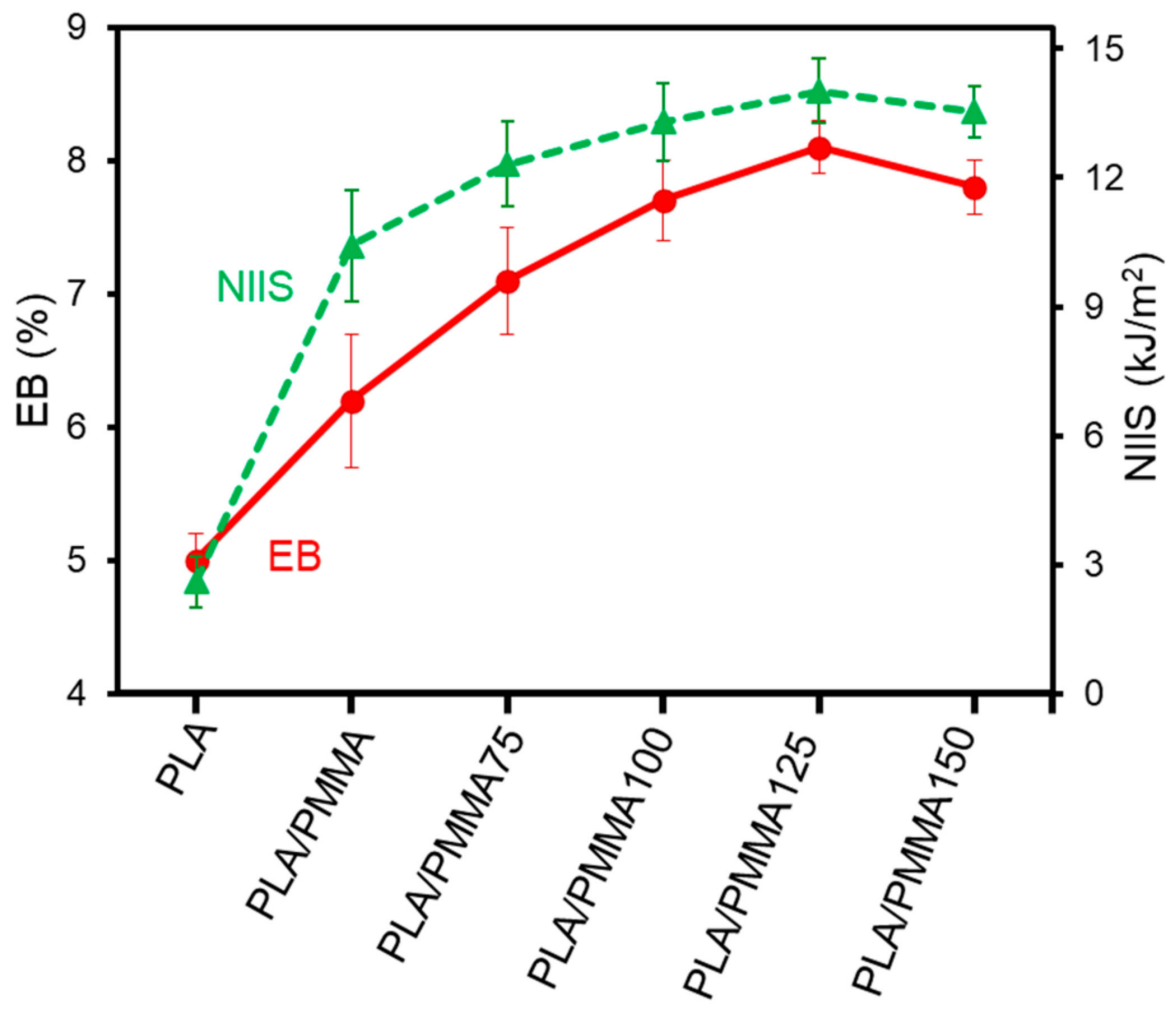
EB and NIIS trends for the fabricated samples.

Figure 2 illustrates the trend of EB values with varying MgO content. The addition of 30 wt% PMMA to neat PLA increases the EB from 5% to 6.2% in the PLA/PMMA sample, likely due to limited exchange reactions between PLA and PMMA chains under high-temperature and shear conditions during melt blending. Enhanced flexibility is further achieved with the addition of 0.075 wt% MgO catalyst in PLA/PMMA75, resulting in an EB value of 7.1%.

The introduction of MgO NPs promotes the formation of PLA-PMMA copolymers, which act as compatibilizers, improving the compatibility between PLA and PMMA chains. This enhanced miscibility persists up to 0.125 wt% MgO, as seen in samples PLA/PMMA100 and PLA/PMMA125, with EB values of 7.7% and 8.1%, respectively. However, at higher MgO concentrations (0.15 wt%), the EB value decreases, likely due to the pronounced chain scission of PLA.

Excessive catalyst concentration facilitates greater PLA chain scission, especially at ester groups, compared to the PLA-PMMA exchange reaction. This leads to the formation of shorter PLA segments, reducing chain entanglement within the polymer matrix. Consequently, poor entanglement results in decreased EB and TS values, as observed in the PLA/PMMA150 sample.

### 3.2. Thermal Behavior

**Figure 3:**
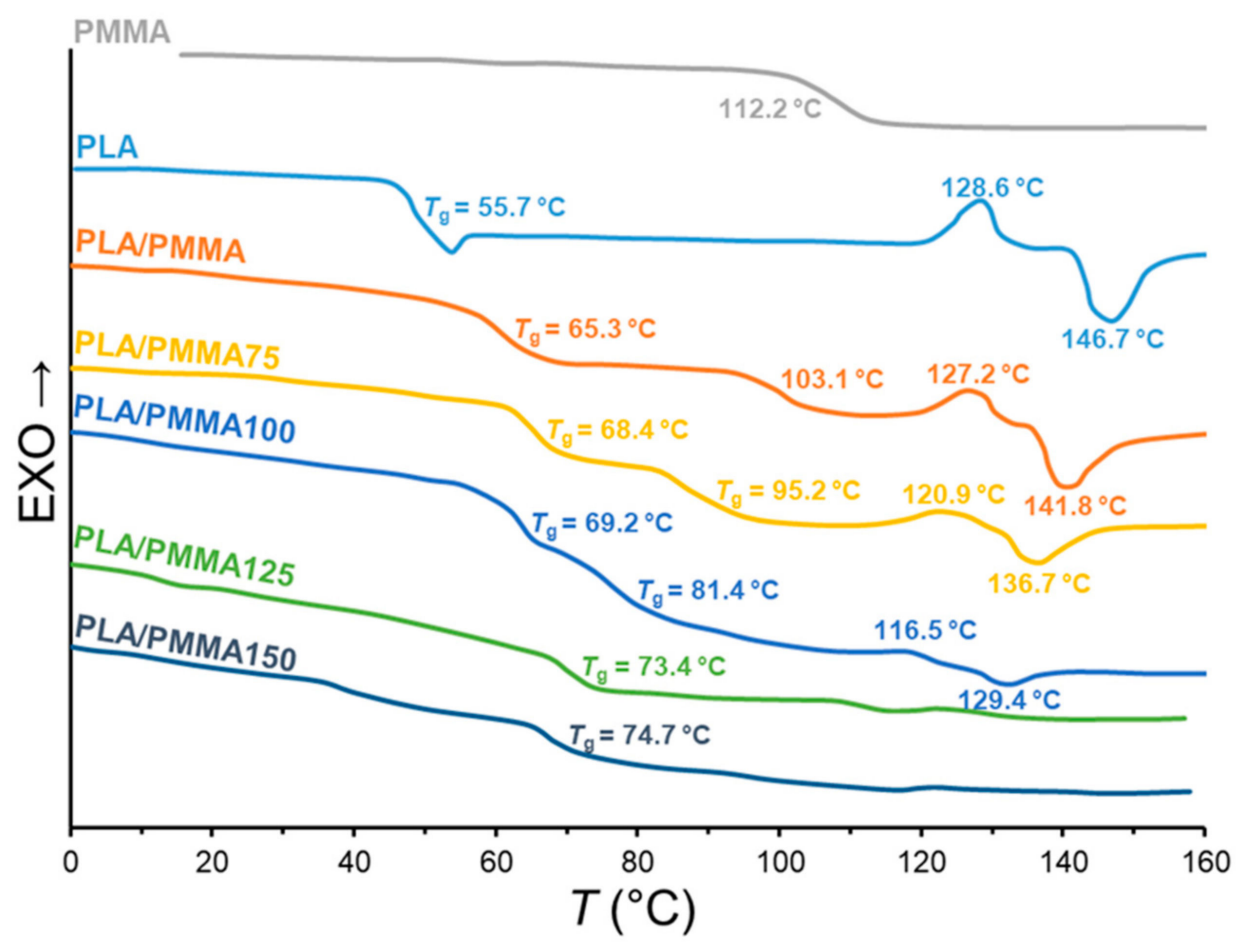
DSC curves of fabricated samples obtained from the second heating cycle.

As previously mentioned, the transesterification reaction between PLA and PMMA is expected to influence the melting and crystallization behavior of PLA/PMMA blends, which can be evaluated using DSC. The DSC curves from the second heating cycle of the fabricated blends are shown in Figure 3, with the relevant characteristic values summarized in Table 2.

The glass transition temperatures (*T*_g_) of pure PLA and pure PMMA, initially 55.7 °C and 112.2 °C, respectively, shift to 65.3 °C and 103.1 °C in the PLA/PMMA blend. Additionally, the cold crystallization temperature (*T*_cc_) and melting temperature (*T*_m_) of neat PLA, originally 128.6 °C and 146.8 °C, respectively, decrease to 127.2 °C and 141.8 °C in the blend. Similarly, the enthalpies of cold crystallization (Δ*H*_cc_) and melting (Δ*H*_m_) of neat PLA, measured at 15.1 J/g and 24.8 J/g, respectively, are reduced to 12.9 J/g and 23.1 J/g. All the samples exhibited low degrees of crystallinity, which decreased with the presence of MgO. This may be the cause of the changes in the mechanical properties of PLA/PMMA blends discussed earlier. These changes can be attributed to partial exchange reactions between PLA and PMMA during melt blending and their incomplete miscibility.

The introduction of 0.075 wt% MgO nanoparticles induces significant changes in the thermal behavior of the PLA/PMMA75 sample. The *T*_g_ values shift further to 68.4 °C and 95.2 °C. Additionally, the *T*_cc_, *T*_m_, Δ*H*_cc_, and Δ*H*_m_ of PLA decrease to 120.9 °C, 136.7 °C, 8.1 J/g, and 15.0 J/g, respectively. These effects are thought to result from the transesterification reactions between PLA and PMMA, which produce copolymers that act as interfacial agents. These copolymers enhance the compatibility of the PLA/PMMA blend and strengthen interphase adhesion, thereby impeding the crystallization process of PLA. Consequently, the Δ*H*_m_ decreases, as polymer crystallinity is directly related to Δ*H*_m_ [26,36]. In conclusion, the produced copolymers with a MgO wt% ≥ 0.100 are non-crystalline.

One notable consequence of block copolymer formation is the reduction in crystal size within the blend matrix. Since the *T*_m_ of a polymer is intrinsically linked to the size of its crystals [26,37,59], it can be inferred that the reduced crystal size in the blend matrix leads to a corresponding decrease in the *T*_m_ value of PLA. This observation further supports the improved miscibility between the two polymers in the presence of a catalyst. Additionally, as the miscibility between polymer phases increases, the glass transition temperatures of the two polymers in the blend matrix converge. This trend is evident when comparing the catalyst-free PLA/PMMA sample with the catalyst-containing PLA/PMMA75 sample.

An increased catalyst concentration of 0.1 wt% in the PLA/PMMA100 sample continues this trend, resulting in more closely aligned *T_g_* values of 69.2 °C and 81.4 °C. The *T*_cc_ and *T*_m_ values of this sample are 116.5 °C and 129.4 °C, respectively, while Δ*H*_cc_ and Δ*H*_m_ have decreased to 2.4 J/g and 6.2 J/g. In the case of the PLA/PMMA125 sample, the best-performing blend, a single *T*_g_ of 73.4 °C was observed, with negligible *T*_cc_ and *T*_m_ values. This result indicates that the crystallization of PLA did not occur within the blend matrix. For the PLA/PMMA150 sample, the behavior closely resembles that of PLA/PMMA125, except for a slightly higher *T*_g_ of 74.7 °C.

The melt flow index (MFI) is a critical property of polymer materials that reflects their flowability in the molten state. This characteristic is closely tied to the molecular structure of polymer chains. Higher MFI values indicate better flowability; within the same polymer type, a higher MFI typically corresponds to lower molecular weight, while a lower MFI suggests higher molecular weight.

**Figure 4:**
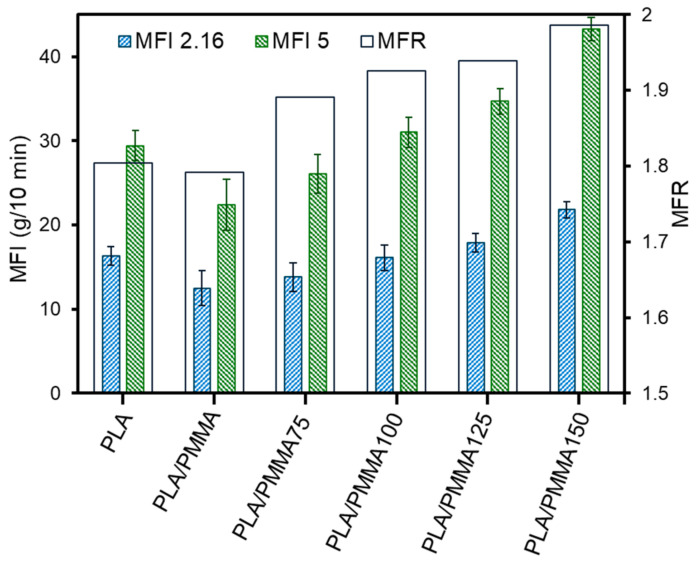
MFI and MFR values for the fabricated samples.

Figure 4 displays the MFI values of the samples tested under two loads: 2.16 kg and 5 kg. Neat PLA exhibits MFI values of 16.3 g/10 min and 29.4 g/10 min under these respective weights. In the PLA/PMMA blend, these values slightly decrease to 12.5 g/10 min and 22.4 g/10 min, respectively. This reduction is attributed to the presence of PMMA, which has lower flowability, thereby slightly diminishing the MFI of the PLA/PMMA blend.

A clear upward trend in MFI values, influenced by the presence of MgO nanoparticles, is observed for both measurement weights. The recorded values are 13.8, 16.1, 17.9, and 21.8 g/10 min at the 2.16 kg measurement weight and 26.1, 31.0, 34.7, and 43.3 g/10 min at the 5 kg measurement weight, corresponding to samples with 0.075, 0.1, 0.125, and 0.15 wt% MgO, respectively. As is well known, longer polymer chains and greater chain entanglements typically lead to a decrease in MFI during the molten phase [3,4]. However, the occurrence of exchange reactions, which are promoted by the MgO catalyst, facilitates the formation of PLA-PMMA block copolymers. This process disrupts PLA chain entanglements in the molten state, resulting in an increase in MFI values. Furthermore, the additional cleavage of PLA chains produces shorter segments, which act as plasticizers, further enhancing the flowability of the blend.

From a statistical perspective, the differences in MFI values among all samples were significant. Notably, the variation in flowability between samples containing 0.125 and 0.15 wt% MgO NPs was statistically significant (*p* < 0.05). In contrast, the differences between samples containing 0.075 and 0.1 wt%, as well as between samples with 0.1 and 0.125 wt% of the catalyst, were negligible (*p* > 0.05). These findings suggest that a higher catalyst concentration (specifically 0.15 wt%) induces substantial PLA chain scission, leading to a more pronounced increase in flowability.

Additionally, the melt flow ratio (MFR), which is the ratio of the MFI at the higher weight to the MFI at the lower weight, serves as an indicator of the polydispersity index (PDI) or the molecular weight distribution (MWD) of polymeric materials [26,60]. The calculated MFR values for the samples are presented in Figure 4. As shown, the presence of PMMA led to a slight decrease in MFR from 1.8 for neat PLA to 1.79 for PLA/PMMA. However, the MFR values for blends with MgO concentrations of 0.075, 0.1, 0.125, and 0.15 wt% were 1.89, 1.93, 1.94, and 1.99, respectively. These values indicate a distinct increase in PDI, particularly between the samples with 0.125 and 0.15 wt% MgO, implying that higher catalyst concentrations result in a higher degree of PLA chain degradation and increased molecular weight distribution heterogeneity. Therefore, these results highlight that 0.125 wt% MgO is the optimal catalyst concentration, as the higher concentration of 0.15 wt% in the PLA/PMMA150 sample causes excessive chain scission.

### 3.3. Wettability Assessment: Water Contact Angle (WCA)

In wettability assessment, the water contact angle (WCA) is defined as the angle formed between a solid sample surface and a droplet of water. This angle provides a quantitative measure of the wettability of different materials and serves as an indicator of the compatibility between the sample surface and water. Surfaces enriched with polar groups exhibit enhanced hydrophilicity, leading to a decrease in the WCA value.

**Figure 5:**
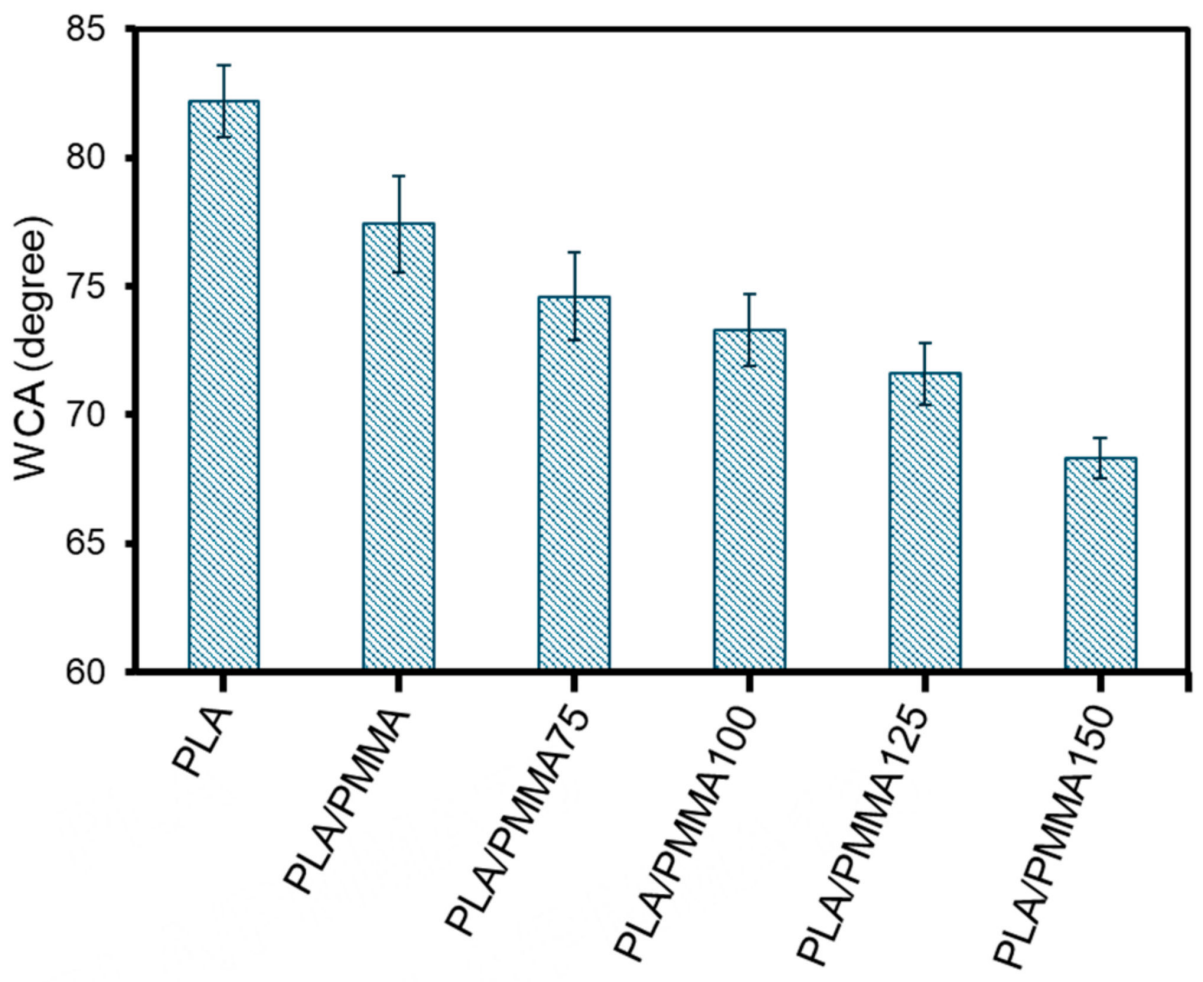
Comparison of the wettability for fabricated blends.

Figure 5 presents the WCA measurements for all samples. The results show that the WCA values for pure PLA and the PLA/PMMA blend are 82.2° and 77.4°, respectively. These values suggest that both PLA and PLA/PMMA surfaces are predominantly hydrophilic, as their WCA values are below 90°.

The introduction of the MgO catalyst resulted in further decreases in WCA values, indicating increased wettability. The WCA values for the samples containing 0.075, 0.1, 0.125, and 0.15 wt% of MgO nanoparticles are 74.6°, 73.3°, 71.6°, and 68.3°, respectively. This phenomenon can be attributed to the increased presence of carboxyl and hydroxyl groups, which form as a result of the cleavage of PLA ester groups and their subsequent migration to the sample surface. In summary, the MgO catalyst accelerates the hydrolysis of PLA chains, promoting the formation of more polar groups on the surface, which in turn enhances the hydrophilicity of the surface.

The statistical analysis for this section revealed a significant difference in the WCA values across all samples. Additionally, a notable difference in wettability was observed between the samples containing 0.125 and 0.15 wt% of catalyst (*p* < 0.05). Conversely, there was no significant difference in the wettability of samples with 0.1 and 0.125 wt% of nanoparticles (*p* > 0.05). These findings further highlight that the scission of PLA chains is intensified with the introduction of 0.15 wt% of catalyst. As previously shown, this intensification negatively impacts the properties of the resulting blends. It is also important to emphasize that the optimum catalyst concentration is 0.125%, which aligns with earlier findings.

### 3.4. SEM, XRD, and TEM Analyses

The analysis of polymer blends conducted through scanning electron microscopy (SEM, Figure S1) revealed that the fracture surfaces of PLA/PMMA samples, catalyzed by MgO NPs, have a homogeneous appearance, devoid of any phase separation. This indicates that these polymer blends demonstrate favorable miscibility.

**Figure 6:**
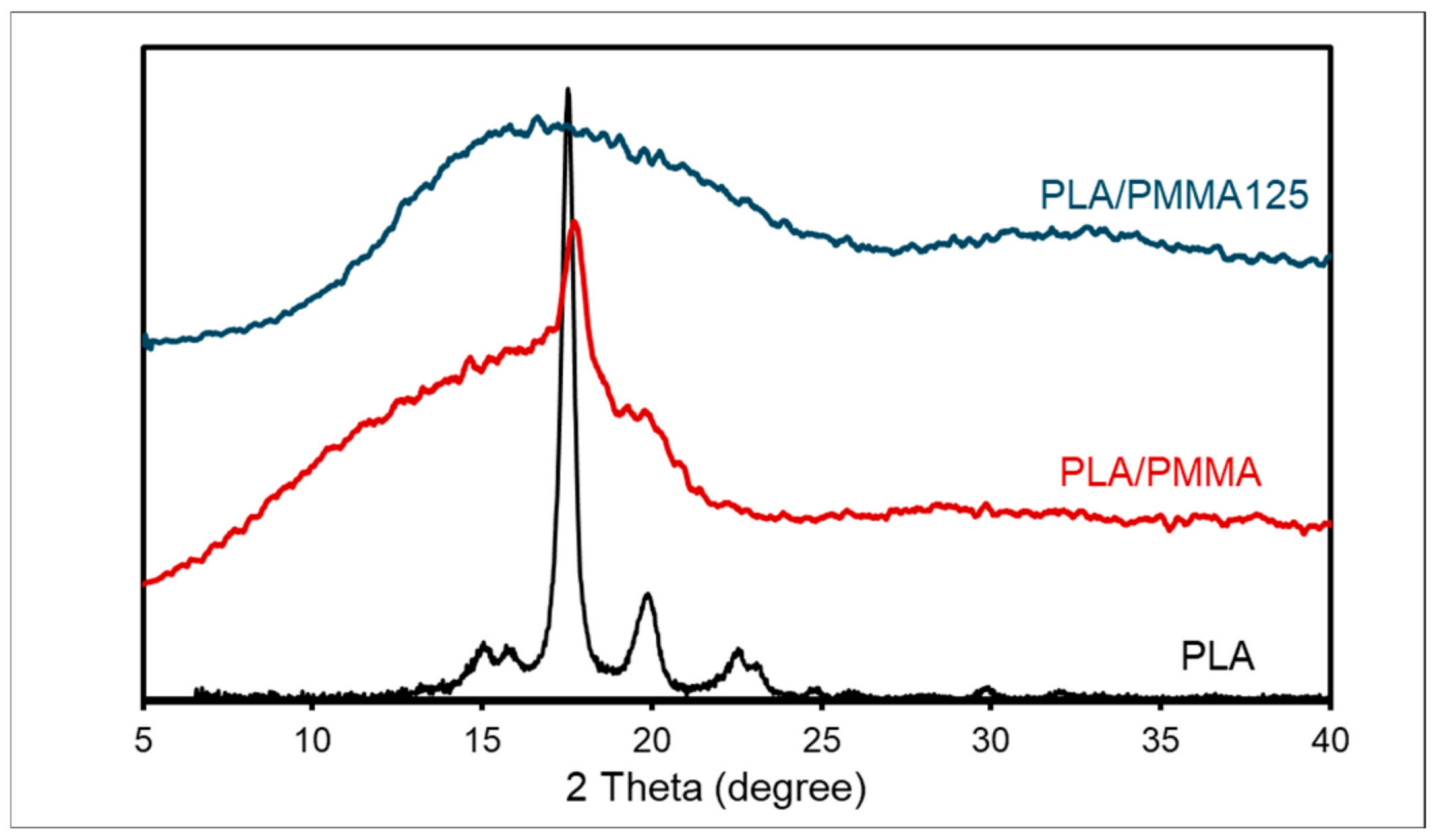
XRD patterns of neat PLA, PLA/PMMA, and PLA/PMMA125.

X-ray diffraction (XRD) was used to analyze the dispersion of nanoparticles (NPs) and evaluate the crystallinity of PLA in the prepared blends. This nondestructive technique is effective in analyzing the presence and classification of crystals in crystalline materials, especially semicrystalline polymers. It also assesses the quality of NP dispersion and evaluates the success of intercalation and exfoliation processes. Figure 6 compares the XRD patterns of PLA, PLA/PMMA, and PLA/PMMA125. The XRD pattern of pure PLA, as shown in the figure, displays distinct peaks at 2θ values of 14–16.5°, 16.5–18.5°, 19–21°, and 22–25°, which correspond to the (104), (200), (014), and (114) lattice planes, respectively [26,36]. These pronounced peaks are indicative of the highly crystalline structure of pure PLA.

The XRD pattern of PLA/PMMA indicates that the melt blending of these two polymers at elevated temperatures and shear stress (without MgO) has reduced PLA crystallinity, as PMMA is recognized as an amorphous polymer. This suggests that even limited exchange reactions between the two polymers can promote some degree of miscibility within the blend. However, the miscibility is not fully achieved, as significant peaks corresponding to the crystalline structure of PLA remain. The introduction of the MgO catalyst in the PLA/PMMA125 sample has resulted in the complete disappearance of all crystalline peaks, indicating that the crystalline structure of PLA has been entirely eliminated. This observation suggests that the compatibility between the two polymers is fully realized. The underlying cause of this is the enhanced formation of PLA-PMMA copolymer chains, which contribute to a consistent alloy matrix, preventing the formation of crystalline PLA structures during the phase transition from liquid to solid. These results are consistent with the findings from DSC.

**Figure 7:**
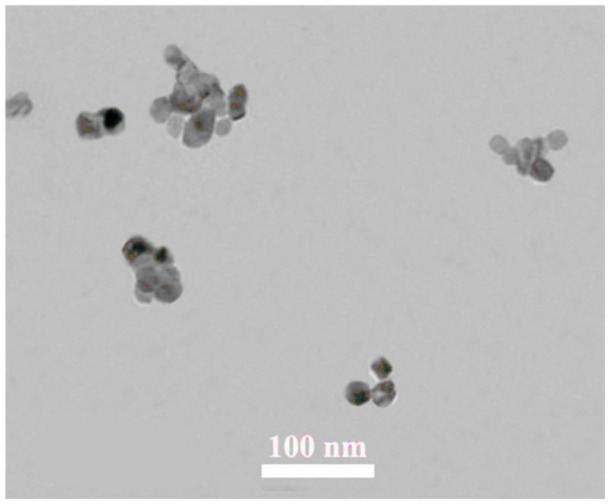
TEM image of PLA/PMMA125 sample.

TEM analysis was performed to conduct a more in-depth examination of the distribution of nanoparticles within the polymer matrix. Using electron beams that penetrate a thin slice of the sample, the TEM instrument captures images that allow for a detailed exploration of the specimen’s internal structure. Due to its remarkable resolution, TEM is widely used in the study of polymeric nanocomposites, effectively revealing the dispersion of nanoparticles within the material. A TEM image of the PLA/PMMA125 sample is shown in Figure 7.

As shown in Figure 7, the MgO NPs have dimensions ranging from 10 to 50 nm. This size range indicates the successful execution of both intercalation and exfoliation processes, where polymer chains penetrate the layers of nanoparticles, followed by their effective separation, layer by layer, during the melt blending process. The beneficial effect of this effective dispersion is evident in the results of exchange reactions observed in previous experiments. This suggests that the appropriate dispersion of NPs within the polymer matrix enhances surface contact and interaction between the NPs and polymer chains, thereby revealing the catalytic effect of MgO. These findings are also supported by the results obtained from the XRD analysis, where the prominent peak at 36.6° is missing.

### 3.5. Cell Culture Assay

The interaction between cells and a material is a critical factor in assessing its biocompatibility or potential cytotoxicity. The physiological process begins with the adsorption of biomolecules onto the material’s surface, followed by cellular interactions. The sequence of cell adhesion and spreading upon contact with materials involves several stages: cell attachment, the development of filopodia, the formation of a cytoplasmic web, the flattening of the cell mass, and the ruffling of the peripheral cytoplasm, all occurring successively. From a tissue engineering perspective, cell adsorption represents a crucial phase, as adhesion occurs before subsequent processes such as spreading, migration, and differentiation [32].

**Figure 8:**
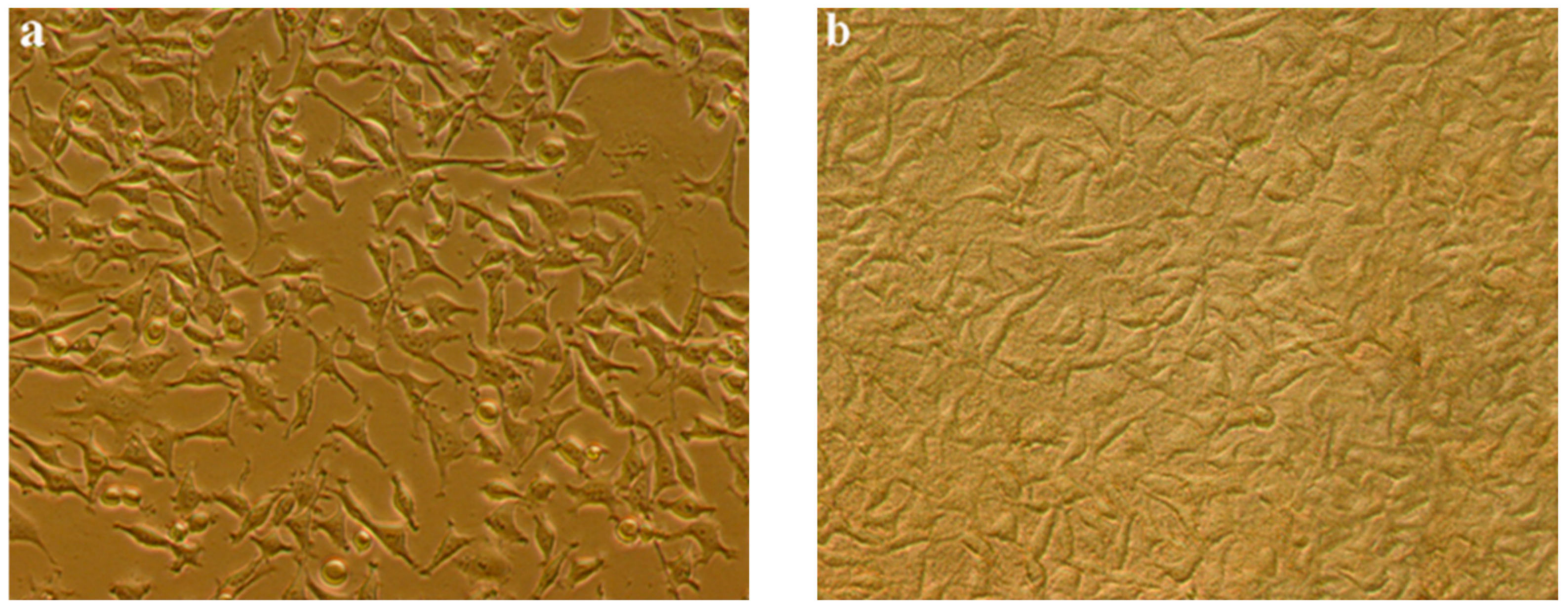
L929 fibroblast cell culture: (**a**) control, (**b**) PLA/PMMA125 (MAG: 100×).

Alternatively, the interaction between fibroblast cells and the material surface is influenced by a specific balance of hydrophilicity/hydrophobicity and the presence of functional groups. Studies have shown that functional groups such as carboxyl and hydroxyl on the material’s surface enhance its hydrophilicity, improving its biocompatibility (i.e., non-cytotoxicity) and promoting cell adhesion [61,62]. Figure 8 presents images of a cell culture on the PLA/PMMA125 sample, along with the control sample. As shown, there is no significant difference between the cells attached and those that have grown on the fabricated sample compared to the control, suggesting that the surface of the sample demonstrates favorable biocompatibility.

**Figure 9:**
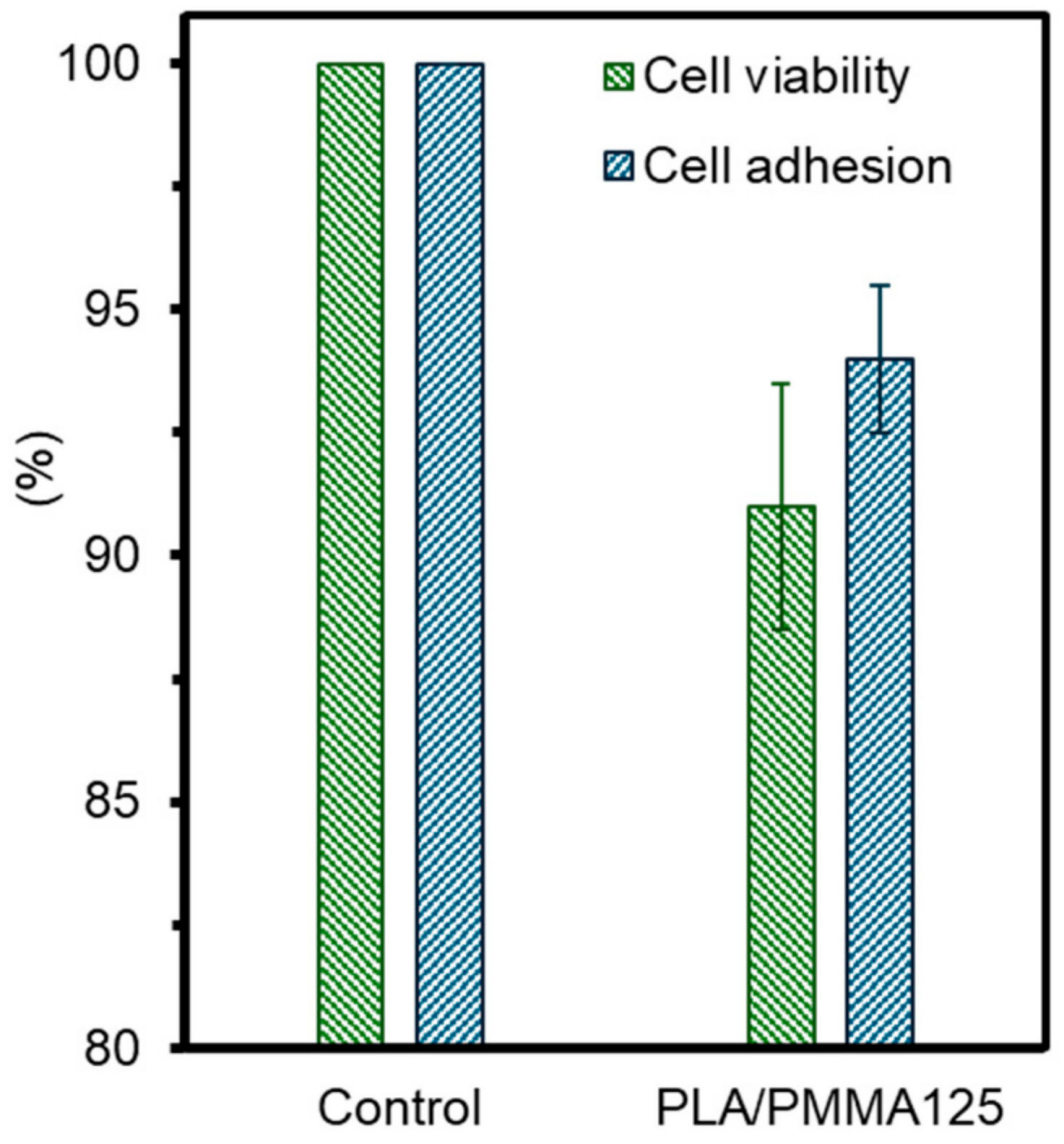
Cell adhesion and cell viability for the sample PLA/PMMA125.

To achieve a quantitative assessment of the results and facilitate better observation, ImageJ software (a Java-based image processing program, version 1.x, developed at the National Institutes of Health, Bethesda, MD, USA) was used to measure the area of adhered cells on the sample surface, compared to the control sample. Assuming that the control represents 100% cell adherence, three micrographs were taken for each sample, and the average adhered-cell area was calculated. As shown in Figure 9, the area of cells adhered to the surface for the sample is 94% of that observed in the control, indicating that the surface possesses adequate biocompatibility.

Additionally, an MTT assay was performed to assess the viability of cells cultured adjacent to the PLA/PMMA125 sample throughout the incubation period (refer to Figure 9). The results revealed that cell viability exceeded 91%, which is considered entirely satisfactory for this sample, as it consists of cytocompatible components and lacks a cytotoxic sol fraction. Furthermore, based on Figure 8 and Figure 9, there is no notable cell debris or morphological alterations, such as cell lysis, and the preservation of spindle shape further affirms the sample’s superior cytocompatibility.

### 3.6. Mechanistic Insights and DFT Investigation

**Scheme 1:**
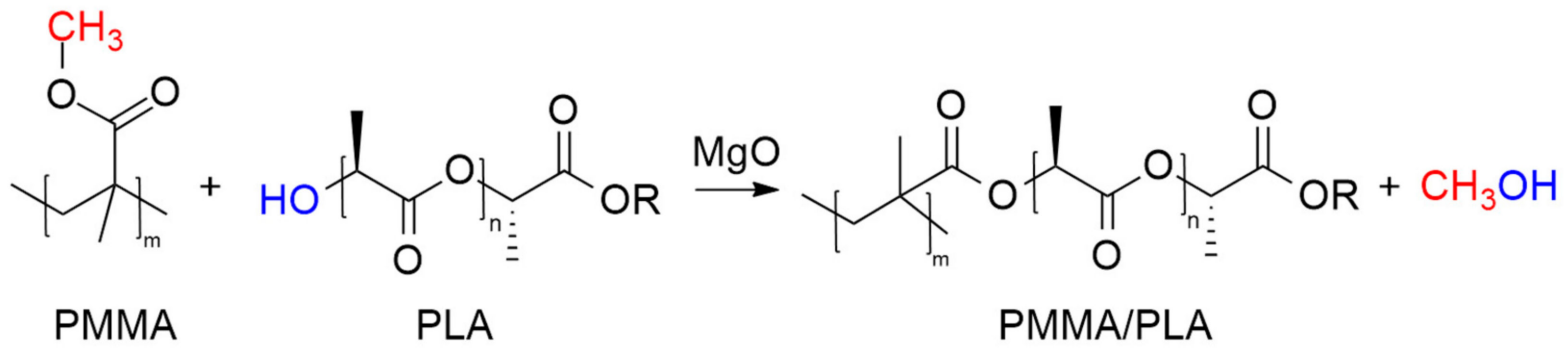
Transesterification between PMMA and PLA giving PMMA/PLA copolymer.

During the melt-blending process, MgO serves a dual function. First, it aids in the chain scission of PLA by breaking its ester bonds under high temperature and shear stress in the molten state. Second, it promotes exchange reactions between the decomposed groups of PLA and the methyl ester groups (MEGs) of PMMA (-COOCH_3_). These reactions lead to the formation of PLA-PMMA block copolymers. Specifically, the main reaction between PLA and PMMA is alcoholysis, where the hydroxyl end groups of PLA interact with the MEGs of PMMA, releasing a methanol molecule. This reaction is depicted in Scheme 1. As transesterification continues, the original homopolymers are gradually converted into block copolymers and, eventually, into random copolymers [38,63].

**Figure 10:**
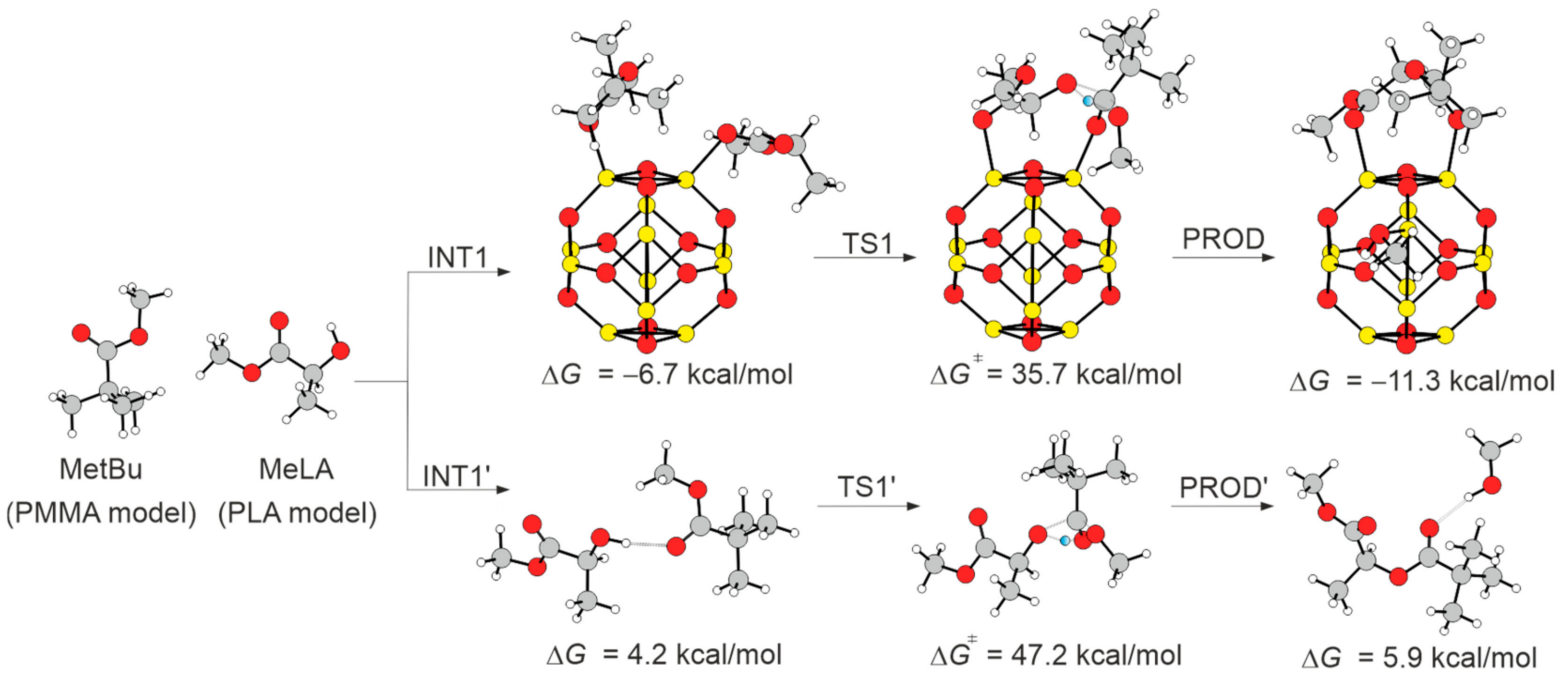
Optimized geometries and calculated Δ*G* for the transesterification reaction, both with (top) and without (bottom) the Mg_12_O_12_ nanocluster. Oxygen is represented in red, magnesium in yellow, carbon in grey, and hydrogen in white.

We performed Density Functional Theory (DFT) static calculations to identify the transition state of the transesterification reaction and to investigate the role of MgO in facilitating these reactions. These methodologies have been extensively validated for similar reaction systems [64,65]. To reduce the computational cost, we modeled the MgO solid as a Mg_12_O_12_ nanocluster [66], the poly (methyl methacrylate) (PMMA) polymer as methyl-tert-butanoate (MetBu), and the poly (lactic acid) (PLA) polymer as methyl-lactate (MeLA). A concerted four-center transesterification transition state (TS) (where nucleophilic attack and MeOH eliminations occur at the same time) was located both with (TS1) and without (TS1’) the Mg_12_O_12_ nanocluster. Both enantiofaces (*re* and *si*) of the carbonyl group were considered, with the *re*-face being always more stable [15,22,67,68]. The results indicate that in the absence of the nanocluster, the reaction is both kinetically and thermodynamically unfavorable (Figure 10).

Specifically, the presence of the Mg_12_O_12_ nanocluster lowers the activation barrier from 47.2 to 42.4 kcal/mol, as calculated relative to the stable coordination intermediate (INT1). In particular, Mg, acting as a Lewis acid, enhances the reactivity of the carbonyl bond in methyl-tert-butanoate, making it more susceptible to transesterification. Furthermore, the Mg_12_O_12_ nanocluster also facilitates the release of methanol, with the products being significantly more stable than the reactants (overall calculated for PROD is Δ*G* = −11.3 kcal/mol). As a final remark, we note that although an activation barrier of 42.4 kcal/mol may seem high, it is consistent with the extrusion conditions used (195 °C). A lower transesterification barrier would compromise the stability of the polymeric materials, as the reaction could occur at room temperature. In contrast, under the extrusion conditions, the reaction is confined to the process itself, preventing undesired reactions from occurring later.

## 4. Conclusions

The incorporation of PMMA in the presence of a transesterification catalyst, MgO, significantly enhanced PLA’s mechanical characteristics. Tensile and NIIS tests revealed that pure PLA is brittle, while the addition of PMMA (with 0.125 wt%. MgO) effectively reduces this brittleness. This enhancement is evident in the EB values during tensile testing and the impact strength observed in NIIS tests. Specifically, pure PLA’s EM and TS decreased from 2.92 and 63 to 2.67 GPa and 55.4 MPa, respectively, for the PLA/PMMA125 sample. Conversely, the EB and NIIS values increased from 5 and 2.6 to 8.1% (an increase of over 60%) and 14.02 kJ/m^2^ (an increase of over 400%), respectively. The DSC technique revealed that the use of the MgO catalyst resulted in the *T*_g_ values of both polymers becoming significantly closer to one another. Additionally, there was a notable reduction and eventual elimination of the cold crystallization of PLA, as well as its *T*_m_ and ∆*H*_m_ values. These results indicate a strong miscibility of the alloy, attributed to the formation of PLA/PMMA block copolymer chains. Furthermore, the MFI analysis revealed a downward trend in viscosity with increasing NP content, while the WCA results indicated the presence of numerous polar groups on the sample surfaces, with a similar downward trend observed as the MgO content increased. XRD and TEM analyses confirmed the effective distribution and dispersion of NPs within the blend matrix. Additionally, XRD analysis revealed a significant reduction in the crystallinity of PLA in the presence of these nanoparticles. These findings collectively demonstrate that the exchange reactions leading to the formation of copolymers from the two polymers have enhanced the compatibility between the polymeric phases in the final alloy. The optimum concentration of the MgO catalyst for achieving the best miscibility was determined to be 0.125 wt%. Furthermore, DFT calculations were conducted to elucidate the role of MgO in the reaction. The results provided valuable insights into the reaction mechanism. The calculations confirmed that the reaction facilitated the transesterification between PLA and PMMA, highlighting the important contribution of MgO in this process. Finally, cell culture assays indicated that the fabricated blend exhibits no cytotoxic effects, confirming the biological safety of all component materials.

## Figures and Tables

**Table 1:** The composition of prepared specimens.

Sample Code	PLA (g)	PMMA (g)	PMMA (wt%)	MgO (g)	MgO (wt%)
PLA	60	-	-	-	-
PLA/PMMA	42	18	30	-	-
PLA/PMMA75	42	18	30	0.045	0.075
PLA/PMMA100	42	18	30	0.060	0.100
PLA/PMMA125	42	18	30	0.075	0.125
PLA/PMMA150	42	18	30	0.090	0.150

**Table 2:** DSC results of fabricated samples obtained from the second heating cycle.

Sample Code	T_g_ (°C) ^1^	T_cc_ (°C) ^2^	T_m_ (°C) ^3^	∆H_cc_ (J/g) ^4^	∆H_m_ (J/g) ^5^	X_c_ (%) ^6^
PLA	55.7	128.6	146.8	15.1	24.8	10.4
PMMA	112.2	-	-	-	-	
PLA/PMMA	65.3 & 103.1	127.2	141.8	12.9	23.1	10.9
PLA/PMMA75	68.4 & 95.2	120.9	136.7	8.1	15.0	7.4
PLA/PMMA100	69.2 & 81.4	116.5	129.4	2.4	6.2	4.1
PLA/PMMA125	73.4	-	-	-	-	
PLA/PMMA150	74.7	-	-	-	-	

^1^ Glass transition temperature, T_g_; ^2^ cold crystallization temperature T_cc_; ^3^ melting temperature, T_m_; ^4^ enthalpy of cold crystallization, ∆H_cc_; ^5^ enthalpy of melting ∆H_m_; ^6^ crystallinity percentage.

## Data Availability

The original contributions presented in this study are included in the article/Supplementary Materials. Further inquiries can be directed to the corresponding authors.

## Supplementary Materials

The following supporting information can be downloaded at https://www.mdpi.com/article/10.3390/polym17070845/s1: Figure S1: SEM picture of the prepared samples: (a) PLA/PMMA75, (b) PLA/PMMA125, (c) PLA/PMMA150 and (d) PLA/PMMA. Scale bar in the pictures is 10 μm; Table S1: xyz and absolute energies (in a.u.) of all DFT computed species.
