# Supplementary material for: PLA/PMMA Reactive Blending in the Presence of MgO as an Exchange Reaction Catalyst
Source: Polymers (Basel). 2025 Mar 21;17(7):845. doi: 10.3390/polym17070845 (PMC11991274; doi:10.3390/polym17070845)
Supplement: Supplementary file 1 [file polymers-17-00845-s001.zip › polymers-3510400-supplementary.pdf]

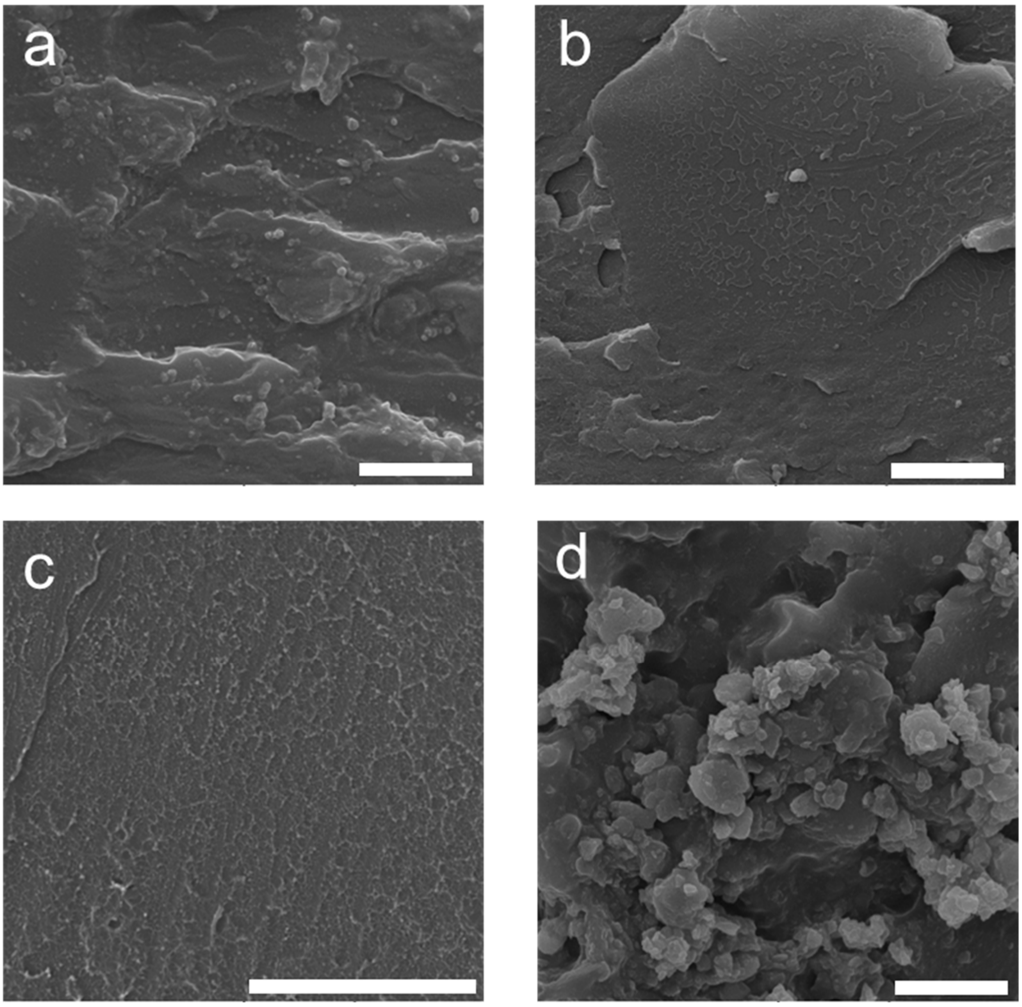

Figure S1. SEM picture of the prepared samples: a) PLA/PMMA75, b) PLA/PMMA125, c) PLA/PMMA150 and d) PLA/PMMA. Scale bar in the pictures is 10 μm.

Table S1. xyz and absolute energies (in a.u.) of all DFT computed species:

20

scf done: -383.0536

C	-2.565725	0.045852	1.591543
H	-2.188316	-0.986638	1.611584
H	-2.226560	0.554220	2.508552
H	-3.666960	0.013207	1.613113
C	-2.087154	0.793671	0.328448
C	-2.620946	2.232912	0.326994
H	-2.277049	2.786694	1.213076
H	-2.275372	2.785527	-0.559164
H	-3.722422	2.226460	0.325959
C	-2.563328	0.044191	-0.934573
H	-2.222421	0.551355	-1.851603
H	-2.185882	-0.988324	-0.952538
H	-3.664520	0.011514	-0.958191
C	-0.553417	0.837682	0.329855
O	0.122351	1.837739	0.329756
O	-0.016296	-0.400783	0.331250
C	1.408436	-0.473988	0.332619
H	1.826124	0.017571	1.224539
H	1.661299	-1.541691	0.333642
H	1.827771	0.016251	-0.559254

24

scf done: -3305,9849

Mg	-1.873042	0.224991	2.259470
Mg	-2.424151	-1.641640	-0.304435
Mg	-0.556344	-2.562231	1.336078
Mg	2.417398	-1.561435	0.617458
Mg	1.285836	-0.265255	2.634097
Mg	0.369406	-2.344946	-1.739020

Mg 1.873711 -0.225136 -2.258984

Mg -1.285637 0.265536 -2.634060

Mg -2.417717 1.561183 -0.617364

Mg 0.556119 2.562529 -1.335645

Mg 2.424472 1.641341 0.303910

Mg -0.370047 2.345047 1.738471

O -0.950710 -2.862169 -0.525232

O 2.145435 -1.818012 -1.211118

O -2.711131 -0.167946 -1.413444

O 0.181495 -0.847900 -2.936176

O 2.140831 1.499021 -1.595920

O -1.130530 2.062865 -1.959460

O 0.950998 2.861721 0.525523

O -2.145780 1.817584 1.211298

O -0.181223 0.848398 2.936117

O 2.711268 0.167714 1.413055

O -2.140964 -1.498629 1.595431

O 1.130306 -2.062631 1.959949

15

scf done: -386.4734

O 2.351786 0.438905 -0.150134

C 1.148399 0.156336 0.353590

C 0.030309 1.008967 -0.240553

O -1.187197 0.643231 0.348238

O 0.944410 -0.696366 1.188624

C 0.294129 2.506688 -0.068290

H 0.020025 0.778704 -1.327430

H 1.221223 2.808380 -0.576792

H -0.548119 3.071898 -0.493542

H 0.368208 2.758554 1.001235

H -0.988222 -0.099478 0.943675

C 3.451805 -0.330246 0.345740

H 3.300924 -1.400534 0.140190

H 4.341915 0.037476 -0.178574

H 3.563195 -0.193963 1.431689

59

scf done: -4075.5665

Mg -4.445641 -1.291825 -0.032571

Mg -2.130548 -2.464064 1.865774

Mg -1.873344 -3.112455 -0.680836

Mg -0.146628 -1.052137 -2.401674

Mg -2.770817 -0.808743 -2.732441

Mg 0.878949 -1.880788 0.612454

Mg 1.146798 0.754977 -0.030371

Mg -0.635694 0.221234 2.627173

Mg -3.255431 0.520004 2.353197

Mg -1.540262 2.570407 0.608529

Mg -1.266117 1.923365 -1.942818

Mg -4.151324 1.257930 -0.684124

O -0.644972 -2.981312 0.780482

O 1.140894 -0.878002 -1.061800

O -2.140120 -0.931179 2.938212

O 0.904077 -0.194186 1.657852

O -0.019315 2.195257 -0.496238

O -1.764367 1.759568 2.279354

O -2.767288 2.497733 -0.867923

O -4.507894 0.352708 0.977856

O -4.272454 -0.361939 -1.711605

O -1.286773 0.388618 -3.000994

O -3.403143 -2.759594 0.447387

O -1.618247 -2.297320 -2.342310

O 2.133193 3.852133 1.331513

C	2.570376	3.251204	0.247043
C	2.840382	4.201952	-0.910607
O	3.212720	3.476148	-2.050088
O	2.815716	2.048754	0.164398
C	4.461052	-1.020228	-1.525370
H	5.000541	-0.237442	-0.970718
H	3.404209	-0.724048	-1.640186
H	4.922346	-1.122446	-2.521046
C	4.533755	-2.380254	-0.786250
C	3.765506	-3.439582	-1.590483
H	2.729508	-3.109232	-1.752119
H	3.744241	-4.409809	-1.070922
H	4.247285	-3.582506	-2.570383
C	5.999189	-2.805299	-0.565867
H	6.064408	-3.767014	-0.031117
H	6.552985	-2.051594	0.012025
H	6.497571	-2.928432	-1.540294
C	3.842936	-2.184800	0.561826
O	2.769851	-2.695037	0.871184
O	4.490519	-1.377505	1.380400
C	3.851473	-1.043340	2.633526
H	2.821929	-0.679106	2.449178
H	4.484415	-0.267017	3.080430
H	3.830089	-1.931510	3.283392
C	1.621308	5.070687	-1.219949
H	0.751971	4.411644	-1.358321
H	1.817158	5.644558	-2.137227
H	1.419573	5.769603	-0.395453
H	3.673821	4.855227	-0.566790
H	3.515283	2.604150	-1.752527
C	1.762067	3.082742	2.491332

H	2.054983	2.030231	2.380708
H	2.252573	3.548422	3.356663
H	0.668377	3.134214	2.604556

59

scf done: -4075.5005

Mg	0.625658	-0.301596	-2.478167
Mg	-0.194475	1.969930	-0.285597
Mg	-0.975252	-0.567869	0.411128
Mg	1.399492	-2.216912	1.944789
Mg	1.220460	-2.806834	-0.618706
Mg	0.982533	0.889187	2.569011
Mg	3.518829	0.127500	2.585816
Mg	2.860719	2.637587	0.683849
Mg	2.688414	2.040749	-1.881429
Mg	4.938980	0.384036	-0.289840
Mg	4.197756	-2.077615	0.349227
Mg	3.149968	-1.084327	-2.531362
O	-0.469096	1.030024	1.408944
O	1.867516	-0.763481	3.020209
O	1.463556	2.800032	-0.620552
O	2.650402	1.829746	2.354394
O	4.771970	-0.557987	1.382274
O	4.149442	2.041129	-0.624283
O	4.570236	-1.199885	-1.325386
O	2.267813	0.574016	-2.958880
O	1.478773	-2.017701	-2.291682
O	2.622595	-3.018902	0.685549
O	-0.690624	0.349132	-1.322055
O	-0.049438	-2.201180	0.681673
O	-3.752632	2.207099	0.889788
C	-3.069183	2.265027	-0.225151

C	-3.510999	1.365472	-1.360833
O	-4.533966	0.420190	-1.015458
O	-2.064319	2.960475	-0.363400
C	-6.670463	-1.211663	-0.035995
H	-6.607553	-1.916145	-0.877503
H	-6.874455	-0.207868	-0.433319
H	-7.524227	-1.511065	0.591922
C	-5.389991	-1.228916	0.814535
C	-5.529328	-0.243317	1.989721
H	-5.703385	0.778843	1.627506
H	-4.621847	-0.238848	2.611068
H	-6.380769	-0.544447	2.619914
C	-5.138352	-2.653114	1.356640
H	-4.225481	-2.690536	1.969093
H	-5.035642	-3.378619	0.535593
H	-5.989151	-2.964829	1.982242
C	-4.124448	-0.851236	0.026234
O	-3.017939	-0.769320	0.534588
O	-4.166942	-1.723481	-1.370600
C	-2.920297	-2.314691	-1.762802
H	-2.188316	-1.550647	-2.068920
H	-3.134294	-3.007146	-2.589162
H	-2.492806	-2.865853	-0.911519
C	-3.992119	2.176487	-2.563456
H	-4.940341	2.690599	-2.342561
H	-4.148486	1.514033	-3.428362
H	-3.231053	2.922569	-2.832796
H	-2.542841	0.853319	-1.604192
H	-4.421342	-0.606026	-1.699625
C	-3.113985	2.769929	2.063143
H	-2.131228	2.273350	2.172866

H	-2.997846	3.856374	1.943929
H	-3.789692	2.543794	2.896033

59

scf done: -4075.5762

Mg	-0.234250	2.476741	-0.465867
Mg	0.998405	-0.504912	-1.289338
Mg	1.142529	0.027451	1.359923
Mg	-1.540781	-0.934768	2.990727
Mg	-1.480507	1.637139	2.383244
Mg	-0.385175	-2.767542	0.679578
Mg	-3.026071	-2.753677	0.769940
Mg	-1.774725	-2.029212	-2.099671
Mg	-1.740199	0.540682	-2.701094
Mg	-4.351685	-0.433776	-1.039476
Mg	-4.217323	0.127459	1.545318
Mg	-2.952162	2.377826	-0.347888
O	1.054450	-1.645378	0.322379
O	-1.655596	-2.581105	2.110028
O	-0.373570	-0.799790	-2.556609
O	-1.751197	-3.172269	-0.622233
O	-4.407529	-1.515828	0.552207
O	-3.160778	-0.736622	-2.442728
O	-4.372985	1.209280	-0.041476
O	-1.745714	2.223725	-1.857181
O	-1.569965	2.784640	0.906463
O	-2.908278	0.414626	2.839450
O	1.029366	1.119396	-0.251624
O	-0.123054	0.325897	2.730274
O	3.307842	-3.297198	-1.315331
C	3.499485	-2.013071	-1.083056
C	4.527055	-1.811111	0.033272

O	4.830061	-0.411180	0.142506
O	2.995343	-1.114456	-1.736422
C	4.141537	2.206962	-0.923293
H	3.064982	2.024495	-1.074777
H	4.715508	1.570817	-1.612615
H	4.369799	3.261303	-1.147803
C	4.508791	1.912143	0.552501
C	6.024805	2.080930	0.784278
H	6.608547	1.420690	0.127067
H	6.301725	1.864210	1.829140
H	6.313183	3.121870	0.569750
C	3.707122	2.842150	1.475637
H	3.890582	2.622792	2.538699
H	2.631569	2.731403	1.275906
H	3.993070	3.887948	1.283922
C	4.103082	0.466709	0.836936
O	3.221731	0.132729	1.612046
O	-0.143072	4.086857	-1.807298
C	-0.596740	5.380803	-1.470647
H	-1.383362	5.355290	-0.691656
H	-0.989048	5.915454	-2.355026
H	0.247745	5.967039	-1.073809
C	4.144478	-2.550845	1.318582
H	3.087094	-2.380659	1.553939
H	4.786415	-2.225226	2.149314
H	4.308207	-3.625558	1.152612
H	5.470540	-2.229614	-0.352887
H	-0.933994	3.480196	-2.125486
C	2.312963	-3.655927	-2.288426
H	1.320315	-3.363757	-1.917876
H	2.508612	-3.154152	-3.246372

H	2.379924	-4.744524	-2.397128

35

scf done: -769.5363

O	3.477309	0.272530	-0.798749
C	2.492629	0.586182	0.074415
C	1.130554	0.232452	-0.519512
O	0.162456	0.451012	0.468357
O	2.695741	1.072096	1.154390
C	-2.686881	-4.439289	-0.553227
H	-2.672049	-4.765054	0.496591
H	-1.648617	-4.416046	-0.921615
H	-3.235601	-5.191252	-1.142337
C	-3.366187	-3.060842	-0.704729
C	-3.374661	-2.629333	-2.177973
H	-2.353676	-2.567773	-2.583066
H	-3.839662	-1.640414	-2.302724
H	-3.941452	-3.358209	-2.778089
C	-4.809306	-3.125170	-0.157473
H	-5.311754	-2.147974	-0.240083
H	-4.823139	-3.431895	0.898242
H	-5.395628	-3.854803	-0.738366
C	-2.581241	-2.029323	0.114401
O	-2.080767	-1.021541	-0.346327
O	-2.510204	-2.357408	1.406230
C	-1.838067	-1.456029	2.299311
H	-1.766126	-1.981946	3.258694
H	-2.427565	-0.534075	2.420329
H	-0.841722	-1.186375	1.923284
C	0.854869	1.043836	-1.793340
H	0.818444	2.118034	-1.553446
H	-0.121388	0.750682	-2.210340

H	1.629284	0.870226	-2.554525
H	1.195574	-0.840035	-0.801012
H	-0.683945	0.095269	0.141403
C	4.807288	0.562967	-0.375347
H	4.931911	1.638624	-0.176528
H	5.056490	0.012171	0.544840
H	5.467116	0.250229	-1.194509

35

scf done: -769.4694

O	-3.421980	-0.836741	-0.866392
C	-2.369907	-0.237880	-0.275430
C	-1.218069	-1.229682	-0.083439
O	-0.173333	-0.594034	0.595947
O	-2.354185	0.929279	0.021952
C	1.497580	1.531975	1.509673
H	2.114908	0.817906	2.071993
H	0.451908	1.417201	1.822082
H	1.831443	2.550855	1.762396
C	1.639928	1.323258	-0.004226
C	0.738614	2.320510	-0.762595
H	-0.316081	2.177964	-0.486837
H	0.838976	2.197999	-1.851580
H	1.034005	3.348987	-0.501449
C	3.113695	1.499280	-0.432335
H	3.236091	1.355833	-1.516556
H	3.763816	0.782329	0.091475
H	3.449576	2.516902	-0.178753
C	1.236085	-0.068440	-0.480062
O	1.139200	-0.495560	-1.581771
O	1.994707	-1.215154	0.653903
C	2.553188	-2.362077	0.044867

H	2.211056	-3.273660	0.563176
H	3.652648	-2.316156	0.105353
H	2.255996	-2.425105	-1.017601
C	-1.662506	-2.501153	0.647976
H	-1.994190	-2.262056	1.670355
H	-0.820886	-3.208079	0.714706
H	-2.487010	-2.990900	0.111997
H	-0.889878	-1.503905	-1.108789
H	0.838251	-1.233450	0.885580
C	-4.550111	-0.006727	-1.144973
H	-4.959033	0.422888	-0.217928
H	-4.270860	0.819314	-1.816583
H	-5.295062	-0.652706	-1.625809

35

scf done: -769.5355

O	3.415639	0.543694	-0.428400
C	2.283085	-0.097646	-0.105675
C	1.331288	0.853794	0.625916
O	0.063478	0.215023	0.776882
O	2.063595	-1.256273	-0.342619
C	-2.145372	-1.154340	1.362965
H	-2.227928	-0.278726	2.024653
H	-1.303164	-1.766016	1.717245
H	-3.068091	-1.746825	1.465253
C	-1.966146	-0.742117	-0.106276
C	-1.823400	-1.998733	-0.999982
H	-0.925673	-2.576108	-0.728070
H	-1.758329	-1.720106	-2.061378
H	-2.704979	-2.645326	-0.864791
C	-3.170418	0.101651	-0.579798
H	-3.070121	0.381572	-1.637702

H	-3.278968	1.017862	0.023711
H	-4.095197	-0.486061	-0.467537
C	-0.696177	0.083620	-0.323971
O	-0.369235	0.582204	-1.382943
O	-1.864881	0.109677	-3.730909
C	-1.390807	0.644792	-4.937051
H	-1.328696	1.752541	-4.928093
H	-2.092015	0.363182	-5.739634
H	-0.391240	0.257236	-5.222601
C	1.837665	1.217730	2.016408
H	1.944674	0.316134	2.638178
H	1.127093	1.898526	2.507340
H	2.813633	1.716777	1.939836
H	1.220361	1.752209	-0.000350
H	-1.258675	0.348171	-3.004984
C	4.410959	-0.226355	-1.108968
H	4.734598	-1.077813	-0.491721
H	4.016624	-0.612818	-2.060501
H	5.251251	0.454114	-1.291910
